# Subtype-Selective Positive Modulation of K_Ca_2.3 Channels Increases Cilia Length

**DOI:** 10.1021/acschembio.2c00469

**Published:** 2022-08-10

**Authors:** Young-Woo Nam, Rajasekharreddy Pala, Naglaa Salem El-Sayed, Denisse Larin-Henriquez, Farideh Amirrad, Grace Yang, Mohammad Asikur Rahman, Razan Orfali, Myles Downey, Keykavous Parang, Surya M. Nauli, Miao Zhang

**Affiliations:** Department of Biomedical and Pharmaceutical Sciences, Chapman University School of Pharmacy, Irvine, California 92618, USA

## Abstract

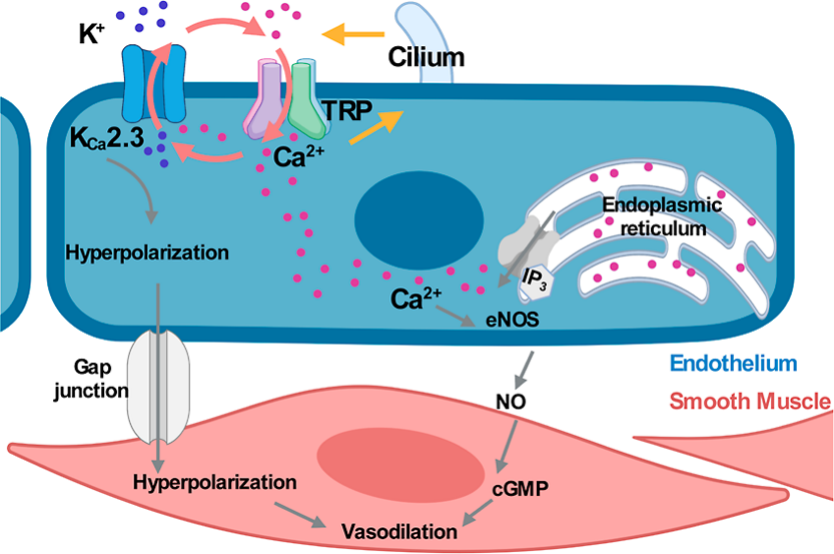

Small-conductance Ca^2+^-activated potassium (K_Ca_2.x) channels are gated exclusively by intracellular Ca^2+^. The activation of K_Ca_2.3 channels induces hyperpolarization, which augments Ca^2+^ signaling in endothelial cells. Cilia are specialized Ca^2+^ signaling compartments. Here, we identified compound **4** that potentiates human K_Ca_2.3 channels selectively. The subtype selectivity of compound **4** for human K_Ca_2.3 over rat K_Ca_2.2a channels relies on an isoleucine residue in the HA/HB helices. Positive modulation of K_Ca_2.3 channels by compound **4** increased flow-induced Ca^2+^ signaling and cilia length, while negative modulation by AP14145 reduced flow-induced Ca^2+^ signaling and cilia length. These findings were corroborated by the increased cilia length due to the expression of Ca^2+^-hypersensitive K_Ca_2.3_G351D mutant channels and the reduced cilia length resulting from the expression of Ca^2+^-hyposensitive K_Ca_2.3_I438N channels. Collectively, we were able to associate functions of K_Ca_2.3 channels and cilia, two crucial components in the flow-induced Ca^2+^ signaling of endothelial cells, with potential implications in vasodilation and ciliopathic hypertension.

## 1 Introduction

Small- and intermediate-conductance Ca^2+^-activated K^+^ (K_Ca_2.x/K_Ca_3.1 or SK/IK) channels are activated exclusively by intracellular Ca^2+^.^1,2^ Four subtypes in the K_Ca_2.x/K_Ca_3.1 channel family are encoded by the *KCNN* mammalian genes: including *KCNN*1 for K_Ca_2.1 (SK1), *KCNN*2 for K_Ca_2.2 (SK2), *KCNN*3 for K_Ca_2.3 (SK3), and *KCNN*4 for K_Ca_3.1 (IK or SK4) channels.

In blood vessels, K_Ca_2.3 and K_Ca_3.1 channel subtypes are often detected on the plasma membrane of endothelial (ET) cells,^3−5^ whereas K_Ca_2.1 and K_Ca_2.2 channel currents are rarely identifiable on the ET cell surface.^6^ K_Ca_2.3 and K_Ca_3.1 channel subtypes seem to have a distinctive distribution and function in ET cells. K_Ca_3.1 channels are often found on the ET cell membrane close to the endoplasmic reticulum (ER) Ca^2+^ store.^7−9^ Ca^2+^ release from the ER triggered by acetylcholine or bradykinin receptors may lead to the opening of K_Ca_3.1 channels nearby.^10^ In contrast, K_Ca_2.3 channels seem to co-localize with mechanosensitive or receptor-operated transient receptor potential (TRP) cation channels.^10,11^ Ca^2+^ influx through these cation channels may activate K_Ca_2.3 channels. The outflow of K^+^ can hyperpolarize ET cells, increase the inward electrochemical gradient for Ca^2+^, and augment the Ca^2+^ influx, which in turn enhances nitric oxide (NO) releases.^12,13^

Non-motile primary cilia are sensory organelles that sense fluid shear stress on the apical membrane of the cells.^14−16^ Fluid flow that produces enough drag force on the top of the cells will bend and activate sensory cilia. Transgenic mouse models with cilia mutations do not survive at birth, confirming the importance of primary cilia in the physiological processes.^17−20^ Primary cilia in vasculatures were once thought to be vestigial organelles and nonfunctional remnants. It has since been shown by different laboratories that cilia are mechanosensory organelles.^21−25^ Cilia in ET cells sense changes in the fluid shear stress and trigger Ca^2+^ signaling and NO releases.^26,27^

Primary cilia have been known as specialized Ca^2+^ signaling compartments.^28,29^ Ca^2+^ influx through TRPM4, TRPV4, TRPC1, polycystic kidney disease 2 (PKD2), and L-type voltage-gated Ca^2+^ (Ca_v_) channels has been considered the main Ca^2+^ source for cilia.^28,29^ Ca^2+^ influx in response to fluid shear stress activates ET K_Ca_2.3 channels.^30^ In ET cells, K_Ca_2.3 channels functionally couple with Ca^2+^-permeable PKD2^11^ and TRPV4^31^ channels and exert a positive feedback influence on intracellular Ca^2+^ signaling.^12,32^ However, it is not clear whether this positive feedback mechanism extends back to the cilia, that is, whether the activation of K_Ca_2.3 channels increases cilia length.

K_Ca_2.3 and K_Ca_2.2a channels have similar amino acid sequences in their cytoplasmic gates, which makes it difficult to develop subtype-selective positive modulators discriminating these two subtypes. We recently identified the binding site of a prototype K_Ca_2.2a/K_Ca_2.3 channel modulator, CyPPA.^33^ We have synthesized a new series of CyPPA analogues.^34^ Here, we report the identification of a subtype-selective K_Ca_2.3 channel modulator, compound **4**, that is ∼21-fold more potent on potentiating human K_Ca_2.3 than rat K_Ca_2.2a channels. The subtype selectivity of compound **4** relies on an I-to-V amino acid residue difference between K_Ca_2.3 and K_Ca_2.2a channels. The pharmacological activation of K_Ca_2.3 channels by compound **4** increased cilia length, whereas the pharmacological inhibition of K_Ca_2.3 channels by AP14145 decreased cilia length in a cultured ET cell line, suggesting the critical role of K_Ca_2.3 channels in the regulation of cilia.

## 2 Results

### 2.1 Compound **4** Subtype Selectively Modulates K_Ca_2.3 Channels

**Figure 1:**
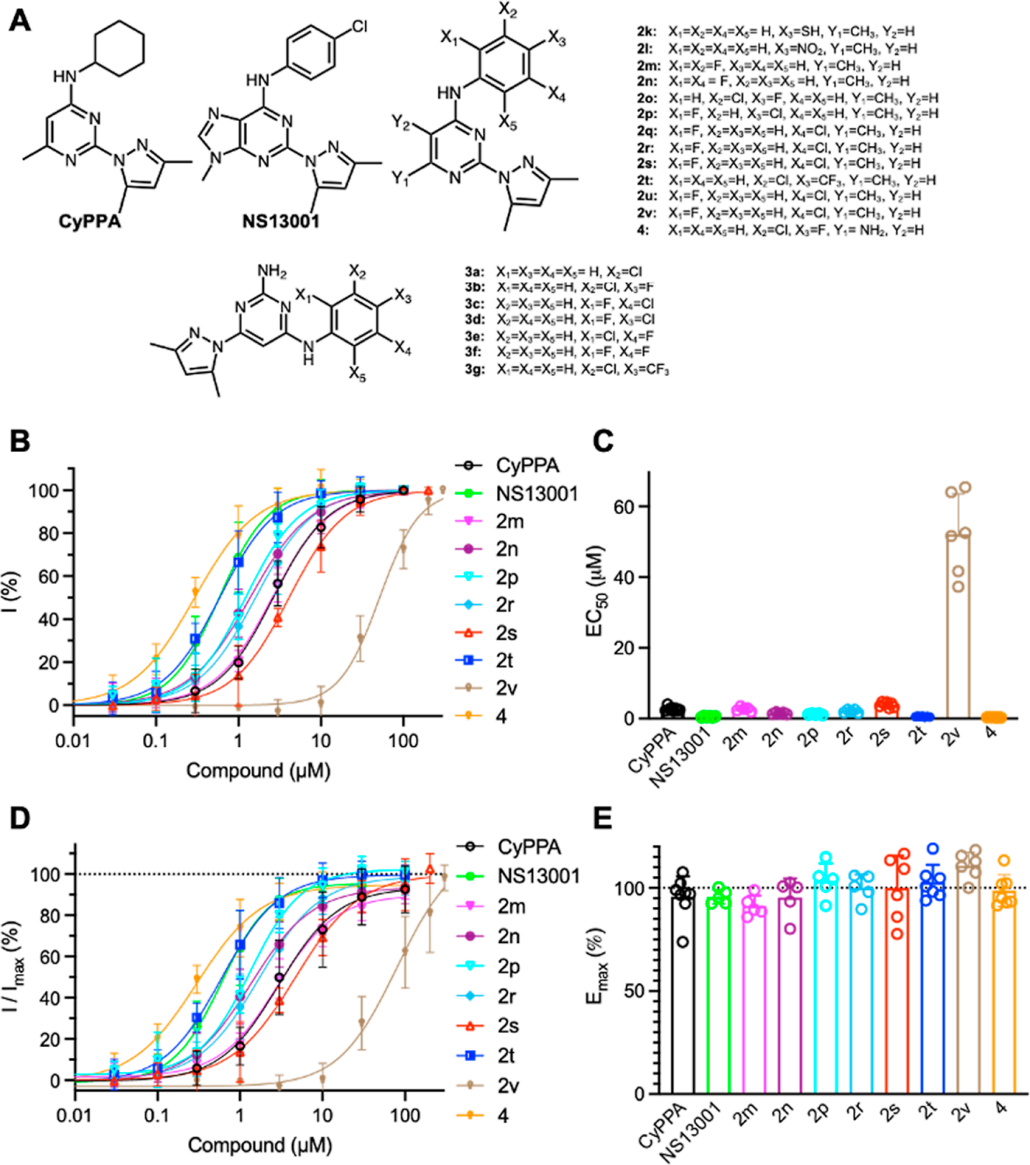
Positive modulation of human K_Ca_2.3 channels by compounds. (A) Chemical structures of compounds **2k–2v**, **3a–3g**, and **4**, compared with those of CyPPA and NS13001. (B) Concentration-dependent potentiation of K_Ca_2.3 channels by compounds. (C) EC_50_ values to compounds of K_Ca_2.3 channels. (D) Responses to compounds of K_Ca_2.3 channels were normalized to the maximal currents induced by 10 μM Ca^2+^. (E) *E*_max_ to compounds of K_Ca_2.3 channels. The numbers of independent recordings are shown in parentheses for CyPPA (8), NS13001 (5), **2m** (5), **2n** (5), **2p** (5), **2r** (5), **2s** (6), **2t** (7), **2v** (6), and **4** (7). Data are presented as mean ± SD.

A series of CyPPA analogues (Figure 1A) were synthesized as described in our previous report.^34^ The potency of these compounds was measured using inside-out patch clamp electrophysiology recordings with human K_Ca_2.3 channels heterologously expressed in HEK293 cells. Positive modulators of K_Ca_2 channels require minimal concentration of Ca^2+^ to be effective.^35^ Therefore, we measured the concentration-dependent responses of the channels to compounds in the presence of 0.15 μM Ca^2+^ (Figure S1). To construct the concentration–response curves, the current amplitudes at −90 mV in response to various concentrations of the compound were normalized to that obtained at the maximal concentration of the compound. The normalized currents were plotted as a function of the compound concentrations. CyPPA, NS13001, and our compounds **2m–2n, 2p, 2r–2t**, **2v**, and **4** concentration-dependently potentiated the activity of K_Ca_2.3 channels (Figure 1B). Among them, NS13001 and compounds **2t** and **4** exhibited submicromolar EC_50_ values (Figure 1C).

The responses induced by 10 μM Ca^2+^ are considered the maximal currents of the K_Ca_2.x channels.^35^ To evaluate the efficacy (*E*_max_) of the compounds on K_Ca_2.3 channels, the current amplitudes at −90 mV in response to the compounds were normalized to that obtained at 10 μM Ca^2+^ [*I*/*I*_max_(%), Figure 1D]. Non-linear regression curve fitting yielded *E*_max_ values for compounds on K_Ca_2.3 channels that are comparable to the *E*_max_ of CyPPA (96 ± 10%, *n* = 8, Figure 1E).

The potency of these compounds on potentiating human K_Ca_2.3 channels is summarized in Table 1 and compared with their previously determined EC_50_ values on rat K_Ca_2.2a channels.^34^ CyPPA and NS13001 exhibited ∼2.7- and ∼4.3-fold selectivity for human K_Ca_2.3 channels over rat K_Ca_2.2a channels (Table 1). Compounds **2t** and **4** are ∼6.3 and ∼21 times more potent, respectively, on potentiating the activity of human K_Ca_2.3 channels than that of rat K_Ca_2.2a channels (Table 1). Among these compounds, compound **4** caught our attention with its ∼21-fold selectivity for human K_Ca_2.3 channels over that of rat K_Ca_2.2a channels (Table 1). We further evaluated the effects of compound **4** on K_Ca_2.1 and K_Ca_3.1 channel subtypes. Compound **4** did not potentiate human K_Ca_2.1 and human K_Ca_3.1 channel subtypes substantively (Figure S2).

**Table 1:** Potency of Compounds on Human K_Ca_2.3 Channels Compared with That on Rat K_Ca_2.2a Channels^a^

compound	EC_50_ on rat K_Ca_2.2a (mean ± SD, μM)	EC_50_ on human K_Ca_2.3 (mean ± SD, μM)
**CyPPA**	7.5 ± 1.6^34^	2.7 ± 0.6
**NS13001**	2.2 ± 0.5^34^	0.50 ± 0.18
**2k**	>100^34^	>100
**2l**	>100^34^	>100
**2m**	5.0 ± 1.1^34^	2.7 ± 0.6
**2n**	1.9 ± 0.4^34^	1.5 ± 0.3
**2o**	1.0 ± 0.2^34^	0.20 ± 0.07^34^
**2p**	2.0 ± 0.3^34^	1.2 ± 0.2
**2q**	0.64 ± 0.12^34^	0.60 ± 0.10^34^
**2r**	3.0 ± 0.7^34^	2.1 ± 0.4
**2s**	3.5 ± 1.0^34^	3.9 ± 0.7
**2t**	3.3 ± 0.8^34^	0.52 ± 0.09
**2u**	>100^34^	>100
**2v**	>30^34^	52 ± 11
**3a**	>100^34^	>100
**3b**	>100^34^	>100
**3c**	>100^34^	>100
**3d**	>100^34^	>100
**3e**	>100^34^	>100
**3f**	>100^34^	>00
**3g**	>100^34^	>100
**4**	6.7 ± 1.6^34^	0.31 ± 0.07

a Some EC_50_ values are reported in ref (34).

### 2.2 Subtype Selectivity of Compound **4** Relies on the HA/HB Helices

**Figure 2:**
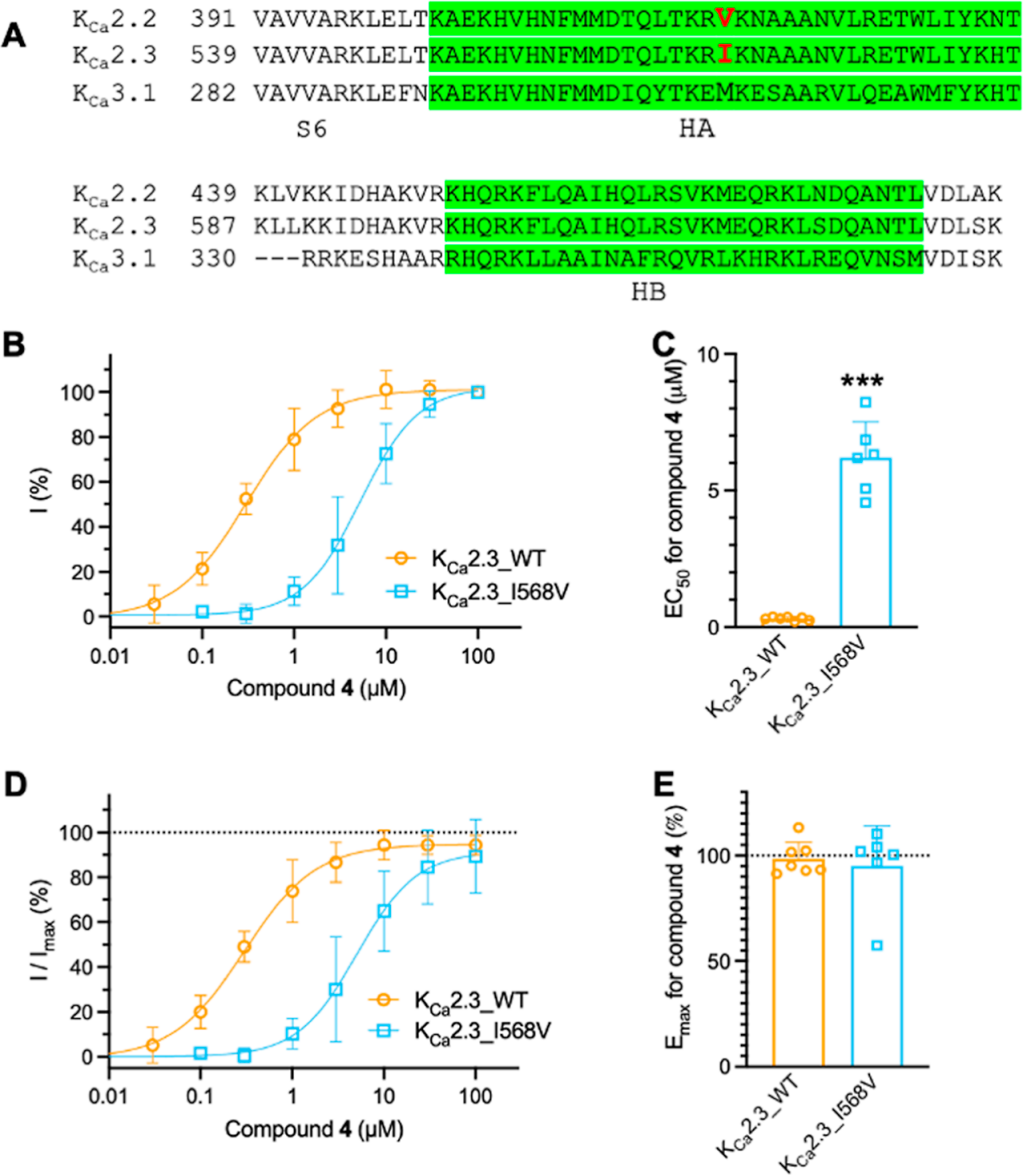
Subtype selectivity of compound 4 relies on the HA/HB helices. (A) Amino acid sequence alignment of rat K_Ca_2.2a [GenBank: NP_062187.1], human K_Ca_2.3 [GenBank: NP_002240.3], and human K_Ca_3.1 [GenBank: NP_002241.1] channels at the proximal C terminus. HA and HB helices are highlighted in green. I568 in K_Ca_2.3 channels and their equivalent residues are shown in bold. (B) Potentiation by compound **4** of the WT and mutant human K_Ca_2.3 channels. (C) EC_50_ values for potentiation by compound **4**. ***P < 0.001 compared with human K_Ca_2.3_WT. (D) Responses to compound **4** were normalized to the maximal currents induced by 10 μM Ca^2+^. (E) *E*_max_ to compound **4** of the WT and mutant K_Ca_2.3 channels. The numbers of independent recordings are shown in parentheses for K_Ca_2.3_WT (7) and K_Ca_2.3_I568V (6). Data are presented as mean ± SD.

Our recent study has revealed that the subtype selectivity of CyPPA for K_Ca_2.2a and K_Ca_2.3 over K_Ca_3.1 channels relies on the HA/HB helices.^33^ We aligned the amino acid sequences of the rat K_Ca_2.2a, human K_Ca_2.3, and human K_Ca_3.1 channel subtypes in the proximal C terminus (Figure 2A). Rat K_Ca_2.2a has a valine residue (V420) equivalent to a methionine residue (M311) of the human K_Ca_3.1 channel in the HA helix. In the HB helix, rat K_Ca_2.2a has a lysine residue (K467), corresponding to an arginine residue (R355) of the human K_Ca_3.1 channel. The V-to-M and K-to-R discrepancies between the amino acid sequences of rat K_Ca_2.2a and human K_Ca_3.1 channels provide an explanation for the subtype selectivity of CyPPA.^33^

We then set out to explore the structural determinants for the ∼21-fold subtype selectivity of compound **4** for human K_Ca_2.3 over rat K_Ca_2.2 channels. Human K_Ca_2.3 has an isoleucine (I568) equivalent to V420 in the HA helix of rat K_Ca_2.2a channels (Figure 2A). The side chain of K_Ca_2.3_I568 would be bulkier than that of K_Ca_2.2a_V420. Thus, the different sizes of a valine (rat K_Ca_2.2a_V420) and an isoleucine (human K_Ca_2.3_I568) may constitute the structural determinants for the subtype selectivity of compound **4**. We tested this hypothesis by mutating K_Ca_2.3_I568 to its corresponding amino acid residue in K_Ca_2.2a, a valine (Figure 2B). The K_Ca_2.3_I568V mutant channel exhibited an EC_50_ value of 6.2 ± 1.3 μM (*n* = 6), which is ∼20-fold less sensitive to compound **4** than the K_Ca_2.3_WT with an EC_50_ value of 0.31 ± 0.07 μM (*n* = 7, Figure 2C). The K_Ca_2.3_I568V mutation did not affect the *E*_max_ values to compound **4**, compared with the K_Ca_2.3_WT channel (Figure 2D,E). The K_Ca_2.3_I568V mutation did not influence the apparent Ca^2+^ sensitivity of K_Ca_2.3 channels (Figure S3A,B).

The corresponding mutation in rat K_Ca_2.2a channels (K_Ca_2.2a_V420I) did not change either the apparent Ca^2+^ sensitivity of K_Ca_2.2a channels (Figure S4A,B) or the *E*_max_ to compound **4** (Figure S4C,D). The K_Ca_2.2a_V420I increased the sensitivity of the channel to compound **4** (Figure S4E,F), corroborating the results acquired from the corresponding K_Ca_2.3_I568V mutation (Figure 2B,C).

### 2.3 Pharmacological Modulation of K_Ca_2.3 Channels Affected Cilia Length

Recently, we identified K_Ca_2.3 channels as the predominant subtype expressed in a mouse ET cell line, whereas the expression of K_Ca_2.1, K_Ca_2.2, and K_Ca_3.1 channel subtypes was not detected by immunoblots.^36^ Thus, we examined whether negative modulation by AP14145 of K_Ca_2.3 channels affected the cilia length of the ET cells. AP14145 inhibited K_Ca_2.3 channels with an IC_50_ value of 0.97 ± 0.39 μM (*n* = 5, Figure S5).

ET cells were incubated with AP14145 (20 μM) for 2 days before cells reached confluency, and the cilia length was evaluated using immunostaining with the antibody of the ciliary marker acetylated α-tubulin (green) and the nuclear marker DAPI (blue, Figure S6A). AP14145 shortened cilia to 2.8 ± 0.1 μm, compared with 6.3 ± 0.3 μm of the solvent control group (Figure S6B,C), suggesting a regulatory role of K_Ca_2.3 channels in the cilia length of ET cells.

**Figure 3:**
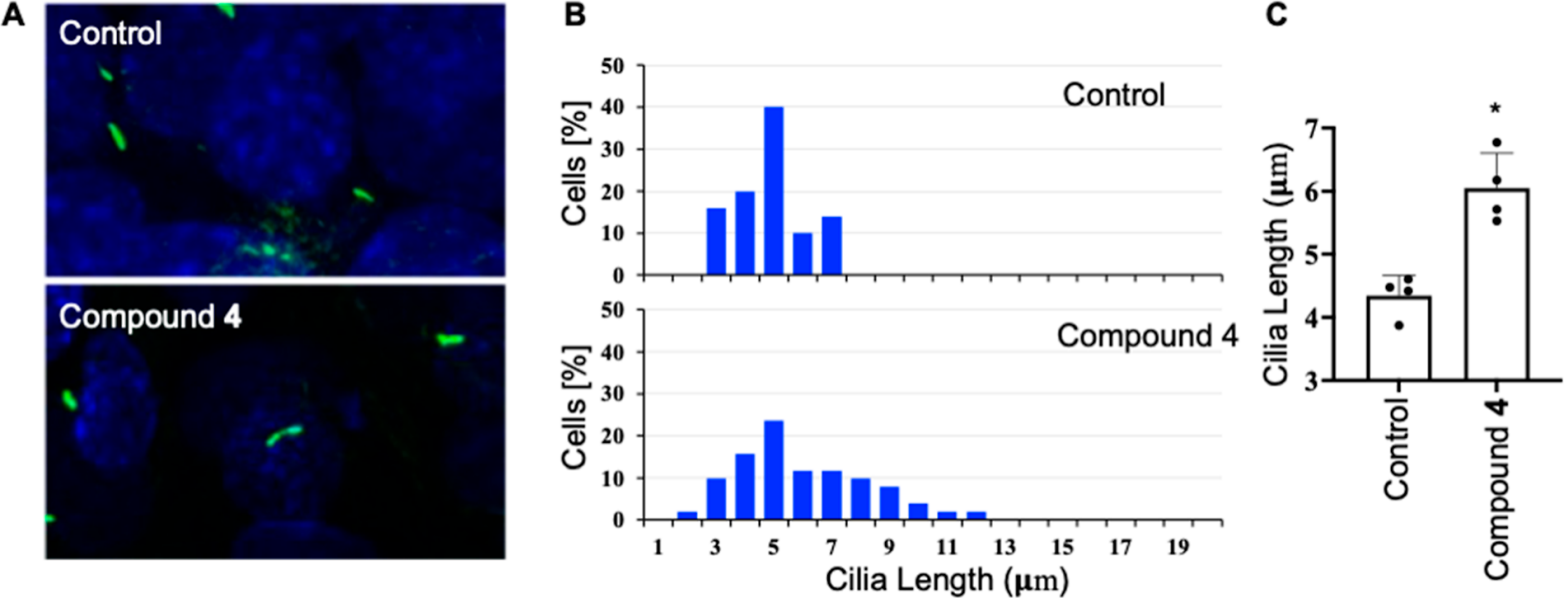
The effects of K_Ca_2.3 channel potentiation by compound 4 on cilia length in ET cells. (A) Cells were stained with the antibody of the ciliary marker acetylated α-tubulin (green) and the nuclear marker (DAPI; blue). (B) Cilia length was grouped in a discreet range, and percent distribution was tabulated. (C) Cilia length is significantly longer in cells treated with the positive modulator, compound **4** (20 μM). *N* = 50–70 for each slide preparation, and a total of four independent slides were used in each group. Data are presented as mean ± SD. *p < 0.05 compared to the control.

Compound **4** potentiated K_Ca_2.3 channels with an EC_50_ value of 0.31 ± 0.07 μM (*n* = 7) (Table 1 and Figure 1C). ET cells were incubated with compound **4** (20 μM) for 2 days before cells reached confluency, and the cilia length was evaluated using immunostaining with the antibody of the ciliary marker acetylated α-tubulin (green) and the nuclear marker DAPI (blue, Figure 3A). Compound **4** increased the cilia length to 6.1 ± 0.6 μm compared with 4.3 ± 0.3 μm of the solvent control group (Figure 3B,C), suggesting potential therapeutic usefulness of K_Ca_2.3 channel positive modulators (e.g., compound **4**) in ciliopathy disease states with abnormal cilia.

To confirm the elongating effect of compound **4** on cilia (Figure 3), an additional ciliary marker Arl13b was used to measure the cilia length (Figure S7A). Also, the γ-tubulin was used as a marker for the basal body (base of a cilium), which cannot be used for the measurement of cilia length. Consistent with the measurements with acetylated α-tubulin (Figure 3), compound **4** (20 μM) increased cilia length (Figure S7B,C). A bee venom toxin, apamin (50 nM),^37^ that selectively blocks K_Ca_2 channels, completely abolished the elongating effect of compound **4** on cilia (Figure S7B,C). The ET cells do not express K_Ca_2.1, K_Ca_2.2, and K_Ca_3.1 channels.^36^ Therefore, the effect of apamin on the ET cells is mediated by the K_Ca_2.3 channel blockade.

### 2.4 Expression of Mutant K_Ca_2.3 Channels Affects Cilia Length

**Figure 4:**
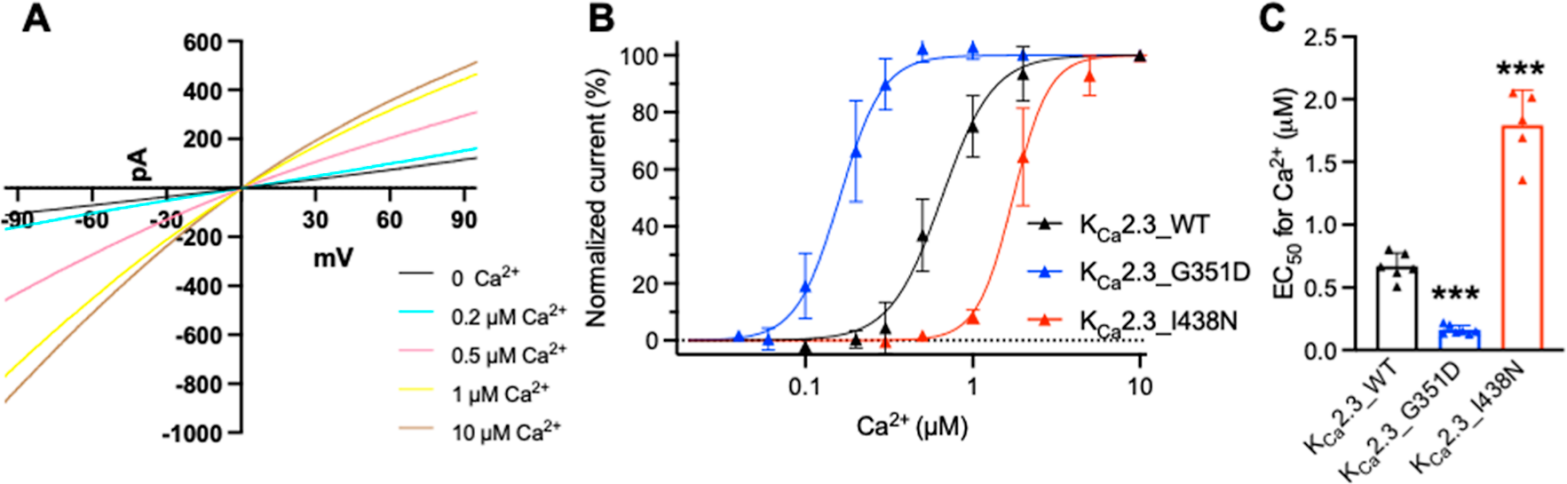
Mutant mouse K_Ca_2.3 channels with altered Ca^2+^ sensitivity. Mutations channels were expressed in ET cells and their apparent Ca^2+^ sensitivity was evaluated using inside-out patch clamp recordings. (A) Representative K_Ca_2.3_WT channel currents in response to Ca^2+^. (B) Concentration-dependent activation by Ca^2+^ of the mutant and WT K_Ca_2.3 channels. (C) EC_50_ values to Ca^2+^ of the mutant and WT K_Ca_2.3 channels. The numbers of independent recordings are shown in parentheses for K_Ca_2.3_WT (6), K_Ca_2.3_G351D (7), and K_Ca_2.3_I438N (5). Data are presented as mean ± SD. ***P < 0.001 compared with K_Ca_2.3_WT.

Positive modulators of K_Ca_2.3 channels potentiate channel activity by increasing the apparent Ca^2+^ sensitivity of the channels,^38^ whereas negative modulators decrease the apparent Ca^2+^ sensitivity of the channels.^39^ To rule out the possibility that compound **4** and AP14145 affected cilia length through their off-target effects other than K_Ca_2.3 channels, we heterologously expressed mutant K_Ca_2.3 channels with altered apparent Ca^2+^ sensitivity (Figure 4). When expressed in ET cells, the K_Ca_2.3 channels exhibited an apparent Ca^2+^ sensitivity of 0.67 ± 0.11 μM (*n* = 6). The G351D mutation significantly increased the apparent Ca^2+^ sensitivity to 0.16 ± 0.04 μM (*n* = 7), while the I438N mutation significantly reduced the apparent Ca^2+^ sensitivity to 1.8 ± 0.3 μM (*n* = 5, Figure 4). Immunoblots (Figures S8A–C) and immunostaining studies (Figure S8D) showed no evidence for different expression levels or localizations of the mutant channels.

**Figure 5:**
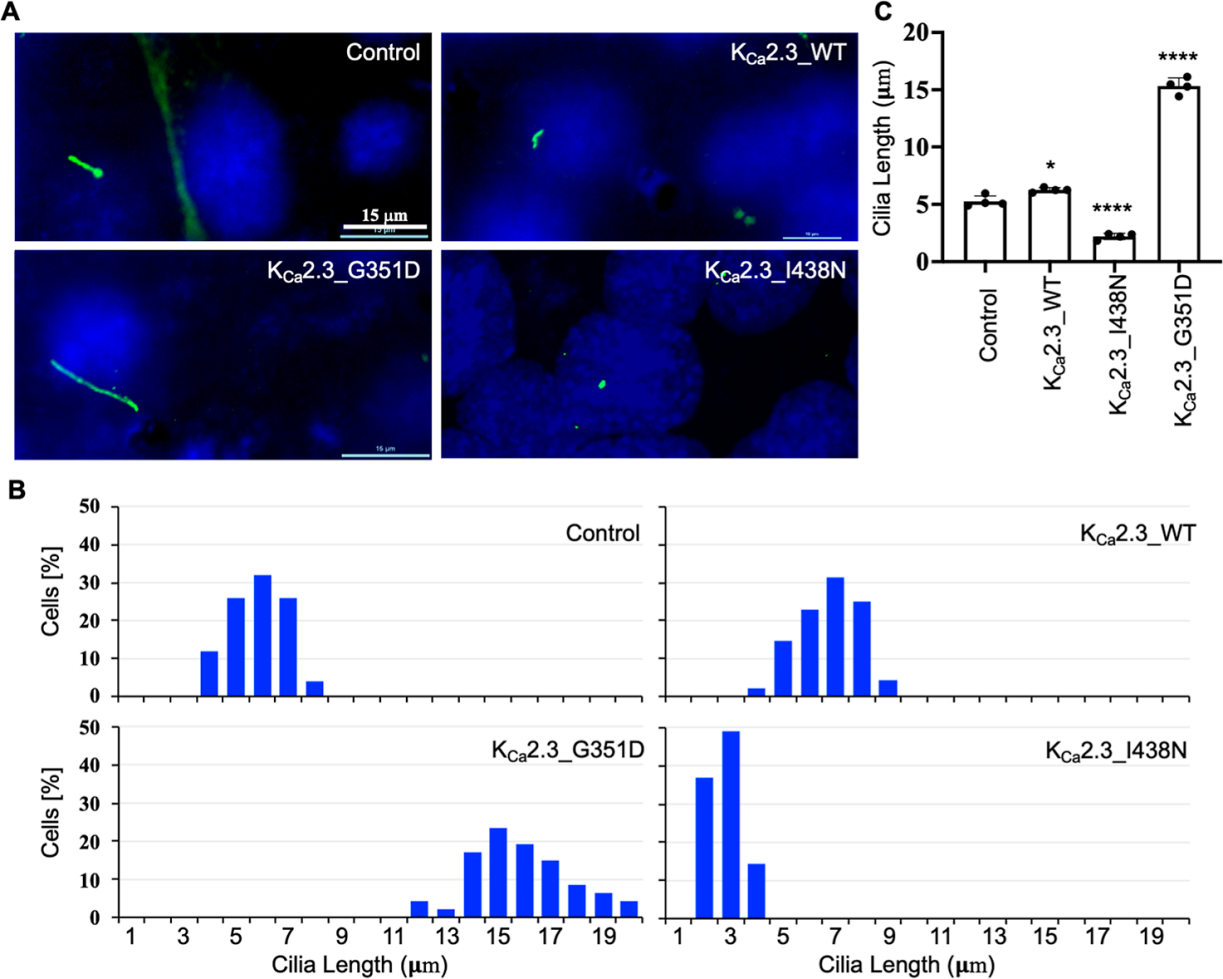
Expression of mouse K_Ca_2.3 channels changes the primary cilia length in ET cells. (A) Cells were stained with the antibody of the ciliary marker acetylated α-tubulin (green) and the nuclear marker DAPI (blue). (B) Cilia length was grouped in a discreet range, and percent distribution was tabulated. (C) Cilia length is significantly longer in cells expressing K_Ca_2.3_WT and K_Ca_2.3_G351D but shorter in cells expressing K_Ca_2.3_I438N channels. *N* = 50–70 for each slide preparation, and a total of four independent slides were used in each group. Data are presented as mean ± SD. *p < 0.05 and ****p < 0.0001 compared to the control.

The higher the apparent Ca^2+^ sensitivity of the mutant channel, the more likely the K_Ca_2.3 channel is opening and then augmenting the Ca^2+^ influx in a positive feedback mechanism. The overexpression of K_Ca_2.3_WT led to a slightly increased cilia length (6.3 ± 0.2 μm) compared with the control (5.3 ± 0.5 μm, Figure 5). K_Ca_2.3_G351D mutant channels with hypersensitivity to Ca^2+^ increased the cilia length even more drastically (15.3 ± 0.7 μm), while the K_Ca_2.3_I438N mutant channels with hyposensitivity to Ca^2+^ reduced the cilia length (2.2 ± 0.3 μm, Figure 5), confirming a role of the K_Ca_2.3 channel in the regulation of cilia length.

### 2.5 Pharmacological Intervention of K_Ca_2.3 Channels Affected Ca^2+^ Signaling

**Figure 6:**
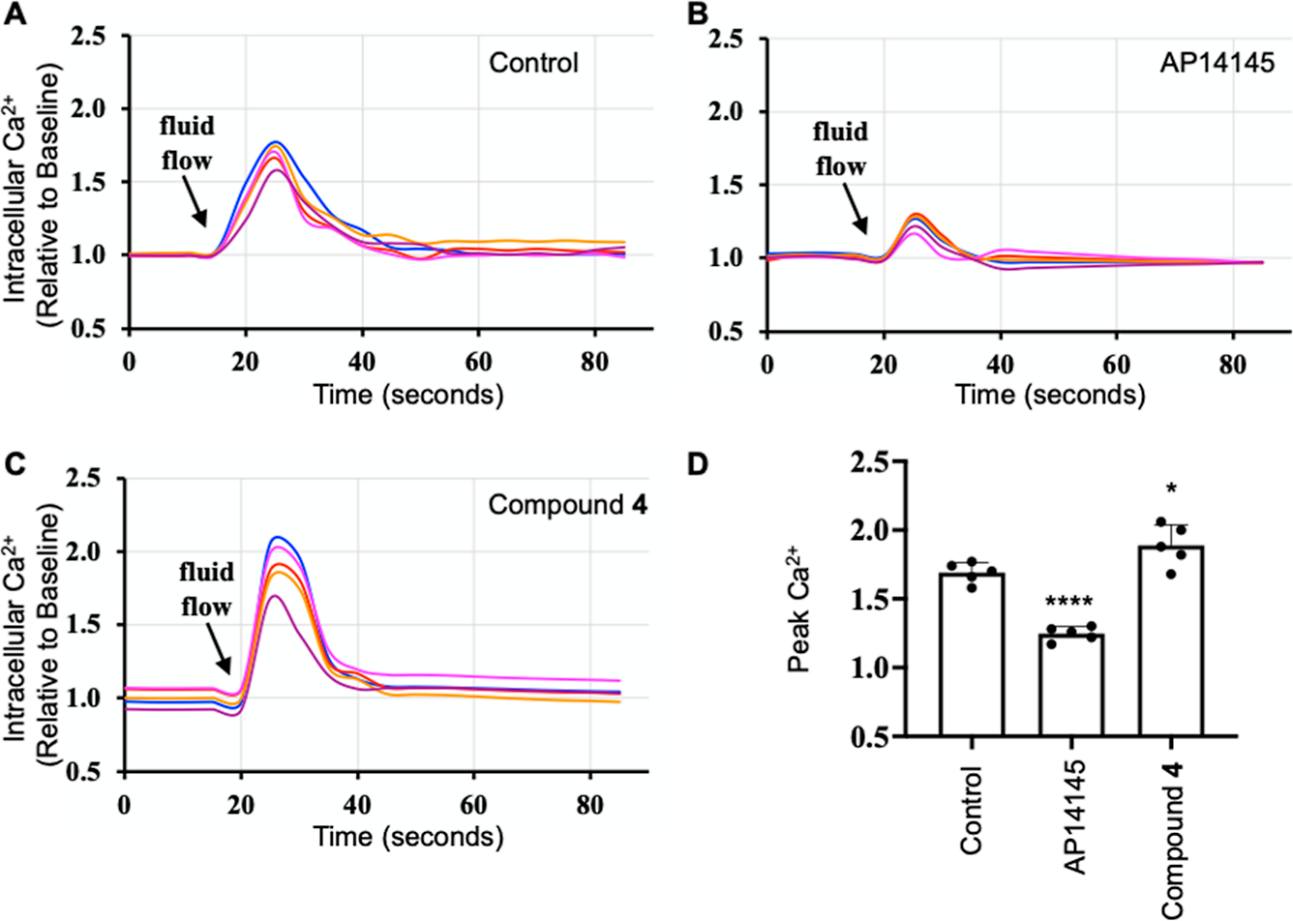
Positive and negative modulation of K_Ca_2.3 channels affected flow-induced cytosolic Ca^2+^ signaling. Fluorescence Ca^2+^ measurements of ET cells treated with (A) solvent control, (B) negative modulator AP14145 (20 μM), and (C) positive modulator compound **4** (20 μM). (D) Peak Ca^2+^ values are significantly increased by compound **4** but reduced by AP14145. The numbers of independent measurements are shown in parentheses for the control (5), AP14145 (5), and compound **4** (5). Data are presented as mean ± SD. *p < 0.05 and ****p < 0.0001 compared with the control.

The opening of K_Ca_2.3 channels induces hyperpolarization, which may increase the inward electrochemical gradient for Ca^2+^ and thus augment the Ca^2+^ influx. Next, we investigated whether the positive modulation or negative modulation of K_Ca_2.3 channels affected the Ca^2+^ signaling, using fluorescence Ca^2+^ imaging (Figure 6). Flow-induced cytosolic Ca^2+^ transients were measured using a ratiometric, high-affinity intracellular Ca^2+^ indicator Fura-2AM. Compared with the control ET cells (Figure 6A), the AP14145-treated ET cells exhibited much weaker Ca^2+^ transients (Figure 6B). In contrast, the compound **4**-treated ET cells exhibited more prominent Ca^2+^ transients (Figure 6C) than the control cells. The significant effects of a negative modulator AP14145 and a positive modulator compound **4** on the flow-induced peak Ca^2+^ values (Figure 6D) suggest a link between the K_Ca_2.3 channel opening and Ca^2+^ signaling, triggered by the shear stress. We have previously generated the non-ciliated IFT88 knockout (KO) mouse ET cells.^40^ Using these cells, we further validate that flow-induced cytosolic Ca^2+^ transients were largely abolished in IFT88 KO ET cells, suggesting the essential role of cilia in flow-induced Ca^2+^ signaling (Figure S9).

## 3 Discussion

Among the four channel subtypes encoded by the mammalian *KCNN* genes, K_Ca_2.3 closely resembles the K_Ca_2.2 channel subtype in pharmacology.^41^ The human K_Ca_2.2a channel does not express as well as the rat K_Ca_2.2a channel, which prevented us from performing inside-out patch clamp experiments. Human and rat K_Ca_2.2a channels are highly homologous, with differences only in the distal cytoplasmic N- and C- termini. In the transmembrane domains and in the cytoplasmic gate including the HA/HB helices (highlighted in green), which CyPPA interacts with, the similarity is 100% (Figure S10). The prototype subtype-selective positive modulator, CyPPA achieved selectivity for K_Ca_2.2 and K_Ca_2.3 channels over K_Ca_2.1 and K_Ca_3.1 subtypes.^35^ CyPPA is also ∼2.7 times more potent on human K_Ca_2.3 than on rat K_Ca_2.2a channels (Table 1). In this study, we identified a positive modulator, compound **4**, that is ∼21-fold selective for human K_Ca_2.3 over rat K_Ca_2.2a channels (Table 1). Compound **4** is largely inactive on human K_Ca_2.1 and human K_Ca_3.1 channels (Figure S2). The significance of this study is not limited to compound **4** itself with an EC_50_ of ∼0.3 μM and a modest subtype-selectivity for human K_Ca_2.3 over rat K_Ca_2.2a channels. The subtype selectivity of compound **4** for human K_Ca_2.3 over rat K_Ca_2.2a channels relies on an I-to-V discrepancy in the HA/HB helices between the two subtypes (Figures 2 and S4), which may offer an opportunity for the development of even more subtype-selective modulators.

The expression of K_Ca_2.3 together with K_Ca_3.1 channels on the plasma membrane of ET cells is well-documented.^3−5^ K_Ca_2.3 channels functionally couple with mechanosensitive and TRP Ca^2+^-entry channels (e.g. PKD2^11^ and TRPV4^31^). We observed a positive feedback effect of K_Ca_2.3 channels on the flow-induced intracellular Ca^2+^ signaling through cilia (Figure 6). Most importantly, the positive feedback extends back to cilia themselves as the positive modulator compound 4 increased the cilia length (Figure 3), while the negative modulator AP14145 reduced the cilia length (Figure S6). These observations allow us to connect K_Ca_2.3 channels and cilia, two crucial components in the flow-induced Ca^2+^ signaling in ET cells, with implications in vasodilation and blood pressure regulation.

The regulation of cilia length by K_Ca_2.3 channel positive and negative modulators (Figures 3 and S6) has been corroborated by the effects on cilial length of the mutant K_Ca_2.3 channels with altered apparent Ca^2+^ sensitivity (Figures 4 and 5). Expression of the Ca^2+^-hypersensitive K_Ca_2.3_G351D mutant channel increased the cilia length, while the Ca^2+^-hyposensitive K_Ca_2.3_I438N mutant channel reduced the cilia length (Figure 5). It is noteworthy that the mouse K_Ca_2.3_G351D mutation used in our study is equivalent to the human K_Ca_2.3_G350D mutation, which causes Zimmermann-Laband syndrome (ZLS).^42^ It has been speculated that during human embryonic development, excessive hyperpolarization due to hypersensitivity to Ca^2+^ of the ZLS-related mutant K_Ca_2.3 channels might result in exaggerated vasodilation in response to shear stress. This in turn might cause edema and vascular ruptures in critical phases of embryonic development, leading to distal digital hypoplasia with aplastic or hypoplastic nails and terminal phalanges.^42^ Our results showed that the equivalent mouse K_Ca_2.3_G351D mutation caused hypersensitivity to Ca^2+^ (Figure 4), which may contribute to vasodilation mediated by the endothelium-derived hyperpolarization.^8,43,44^ Our finding here that the expression of K_Ca_2.3_G351D mutant channels increased cilia length in ET cells (Figure 5) could also be translated into increased sensitivity and vasodilation in response to blood flow. Both of these two mechanisms might underlie the vasodilation and vascular rupture speculated in the embryonic development of ZLS patients, although further studies will be needed to elucidate the developmental biology.

We and other laboratories have previously reported that rapamycin increases cilia length in epithelial cells, resulting in the inhibition of cell proliferation.^45,46^ On the other hand, rapamycin-induced cilia length increase correlates to an elevated response to fluid shear stress in ET cells.^47^ The function of primary cilia as mechanosensory organelles depends on the length of cilia; lengthening primary cilia enhance cellular mechanosensitivity.^48,49^ Dopamine, for example, also increases cilia length and function, resulting in enhanced cellular mechanosensitivity.^50^ While dopamine specificity was a concern, drugs that improve sensory cilia function by elongating cilia length have been coined “ciliotherapy”.^51^ A more specific cilia-targeted therapy in ET cells has also been proposed to remedy hypertension.^52,53^ We therefore are hopeful that subtype-selective positive modulators of KCa2.3 channels (e.g., compound **4**) would have a great potential to be a potential ciliotherapy.

## 4 Experimental Section

### 4.1 Materials

Materials are listed in Table 2.

**Table 2:** 

reagent or resources	source	identifier
Chemicals		
CyPPA	Alomone Labs	C-110
NS13001	ChemShuttle	104258
compound **2k**	in-house synthesis^34^	N/A
compound **2l**	in-house synthesis^34^	N/A
compound **2m**	in-house synthesis^34^	N/A
compound **2n**	in-house synthesis^34^	N/A
compound **2o**	in-house synthesis^34^	N/A
compound **2p**	in-house synthesis^34^	N/A
compound **2q**	in-house synthesis^34^	N/A
compound **2r**	in-house synthesis^34^	N/A
compound **2s**	in-house synthesis^34^	N/A
compound **2t**	in-house synthesis^34^	N/A
compound **2u**	in-house synthesis^34^	N/A
compound **2v**	in-house synthesis^34^	N/A
compound **3a**	in-house synthesis^34^	N/A
compound **3b**	in-house synthesis^34^	N/A
compound **3c**	in-house synthesis^34^	N/A
compound **3d**	in-house synthesis^34^	N/A
compound **3e**	in-house synthesis^34^	N/A
compound **3f**	in-house synthesis^34^	N/A
compound **3g**	in-house synthesis^34^	N/A
compound **4**	in-house synthesis^34^	N/A
Fura2-AM	Thermo Fisher Scientific	F-1221
DAPI	Southern Biotech	0100-20
Antibodies		
fluorescein secondary antibody	Vector Labs Burlingame	FI-2000
anti-acetylated α-tubulin	Sigma-Aldrich	T-7451
anti-GFP	Novus Biological	NB600-308SS
anti-GAPDH	Abcam	ab181602
anti-Arl13b	Proteintech	17711-1-AP
anti-γ-tubulin	Proteintech	15176-1-AP
Experimental Models: Cell Lines		
Human: HEK293	ATCC	CRL-11268
Mouse: ET	in-house^26,27^	N/A
Mouse: IFT88 KO	in-house^40^	N/A
Recombinant DNA		
pcDNA3.1(+)	Thermo Fisher Scientific	V79020
pIRES2-AcGFP1	Takara Bio	632435
Software and Algorithms		
GraphPad Prism 9.0.2	GraphPad Software Inc.	RRID: SCR_002798
Clampfit 10.5	Molecular Devices	RRID: SCR_011323
pClamp 10.5	Molecular Devices	RRID: SCR_011323
Clustal Omega server	https://www.ebi.ac.uk/Tools/msa/clustalo/	RRID: SCR_001591

### 4.2 Electrophysiology

The effect of compounds on the K_Ca_2.x/K_Ca_3.1 channels was investigated as previously described.^54,55^ Briefly, the rat K_Ca_2.2a, human K_Ca_2.1, human K_Ca_2.3, or human K_Ca_3.1 channel cDNA constructs were either generated in-house or through molecular cloning services (Genscript, Piscataway, NJ, USA). These channel cDNAs in the pIRES2-AcGFP1 vector, along with calmodulin cDNA in the pcDNA3.1 + vector, at a ratio of 10:1 (ORF ratios), were transfected into cells using the calcium–phosphate method. K_Ca_ currents were recorded 1–2 days after transfection using an Axon200B amplifier (Molecular Devices, San Jose, CA) at room temperature. The resistance of the patch electrodes ranged from 3 to 5 MΩ. The pipette solution contained the following (in mM): 140 KCl, 10 Hepes (pH 7.4), and 1 MgSO_4_. The bath solution containing (in mM) 140 KCl, 10 Hepes (pH 7.2), 1 EGTA, 0.1 Dibromo-BAPTA, and 1 HEDTA was mixed with Ca^2+^ to obtain the desired free Ca^2+^ concentrations, calculated using the software written by Chris Patton (https://somapp.ucdmc.ucdavis.edu/pharmacology/bers/maxchelator/webmaxc/webmaxcS.htm). The Ca^2+^ concentrations were verified using a Ca^2+^ calibration buffer kit (Thermo Fisher Scientific). Briefly, a standard curve was generated using the Ca^2+^ buffers from the kit and a fluorescence Ca^2+^ indicator. Then, the Ca^2+^ concentrations of the bath solution were determined through interpolation on the standard curve.

High-resistance seals (>1 GΩ) were formed before inside-out patches were obtained. The seal resistance of inside-out patches was >1 GΩ, when the intracellular face was initially exposed to a zero-Ca^2+^ bath solution. Currents were recorded by repetitive 1-*s*-voltage ramps from −100 to +100 mV from a holding potential of 0 mV. The currents were filtered at 2 kHz and digitized at a sampling frequency of 10 kHz. At the end of the experiment, the integrity of the patch was examined by switching the bath solution back to the zero-Ca^2+^ buffer. Data from patches, which maintained the seal resistance (>1 GΩ) after solution changes, were used for further analysis.

To measure the effect of the positive modulators, the intracellular face was exposed to bath solutions with 0.15 μM Ca^2+^. One minute after the switching of bath solutions, 10 sweeps with a 1 s interval were recorded at a series of concentrations of the compound in the presence of 0.15 μM Ca^2+^. The maximal K_Ca_2.x/K_Ca_3.1 current in response to 10 μM Ca^2+^ was then recorded.

To measure the effect of the negative modulator Ap14145, the intracellular face was exposed to bath solutions with 0.5 μM Ca^2+^. One minute after the switching of bath solutions, 10 sweeps with a 1 s interval were recorded at a series of concentrations of AP14145 in the presence of 0.5 μM Ca^2+^.

### 4.3 Cilia Measurements

Cilia length was measured by direct immunofluorescence for the cilia marker with anti-acetylated α-tubulin or Arl13b staining. The cells were fixed for 10 min (4% paraformaldehyde/2% sucrose in PBS) and permeabilized for 5 min (10% Triton X-100). Acetylated α-tubulin (1:10,000 dilution, Sigma-Aldrich, St. Louis, MO) or Arl13b (1:50 dilution, Proteintech, Rosemont, IL) and fluorescein isothiocyanate-conjugated (1:1000 dilution, Vector Labs Burlingame, CA) antibodies were each incubated with the cells for 1 h at 37 °C. Microscope slides were then mounted with DAPI (Southern Biotech, Birmingham, AL) hard set mounting media. A Nikon Eclipse Ti-E inverted microscope with NIS-Elements imaging software (version 4.30) was used to capture the images of primary cilia. Automated image acquisition was conducted in 100× magnification fields. Cilia length analysis followed a standard calculation as previously described.^56^

### 4.4 Flow-Induced Ca^2+^ Measurements

Cells were loaded with 5 μM Fura2-AM (Thermo Fisher Scientific, Waltham, MA) at 37 °C for 30 min. Cells were then washed with Dulbecco’s phosphate-buffered saline and observed under a 40× objective lens using a Nikon Eclipse Ti-E microscope controlled by Elements software. Cytosolic calcium was observed by recording Ca^2+^-bound Fura excitation fluorescence at 340/380 nm and emission at 510 nm. Baseline Ca^2+^ was observed for 5 min prior to data acquisition. Fluid shear stress was then applied to cells utilizing an Instech P720 peristaltic pump with an inlet and outlet setup. The fluid was perfused on the glass-bottom plates at a shear stress of 5 dyn/cm^2^. After each experiment, the maximum calcium signal was obtained with ATP (10 μM) to confirm cell viability. Conditions for all experiments were maintained at 37 °C and 5% CO_2_ in a stage top cage incubator (okoLab, Burlingame, CA). Ca^2+^ analysis followed a standard calculation as previously described.^56^

### 4.5 Immunoblots

The protein concentrations in ET cell lysates were determined using a BCA protein assay kit (Thermo Fisher Scientific, Waltham, MA). Equal amounts of protein (15 μg) were separated by sodium dodecyl sulfate–polyacrylamide gel electrophoresis gel (Bio-Rad Laboratories, Hercules, CA). The proteins were transferred to polyvinylidene fluoride (PVDF) membranes and incubated overnight at 4 °C with the primary GFP antibody (1:2000; Novus Biological, Centennial, CO) or GAPDH antibody (1:5000; Abcam, Waltham, MA). The PVDF membranes were washed with Tris-buffered saline (0.1% Tween 20) and incubated with the anti-rabbit antibody (1:3000; cell signaling technology, Danvers, MA) as the secondary antibody for 1 h at room temperature and then washed with Tris-buffered saline (0.1% Tween 20). The chemiluminescence signals were detected on a ChemiDoc XRS system (Bio-Rad Laboratories, Hercules, CA) after incubation with Luminol/enhancer solution (Thermo Fisher Scientific, Waltham, MA). Densitometry analyses were performed using the ImageJ computer program.

### 4.6 Data and Statistical Analysis

Patch clamp recordings were analyzed using Clampfit 10.5 (Molecular Devices LLC, San Jose, CA), and concentration–response curves were analyzed in GraphPad Prism 9.0.2 (GraphPad Software Inc., La Jolla, CA). To construct the concentration-dependent potentiation of channel activities by the compound, the current amplitudes at −90 mV in response to various concentrations of the compound were normalized to that obtained at a maximal concentration of the compound. The normalized currents were plotted as a function of the concentrations of the compound. EC_50_ values and Hill coefficients were determined by fitting the data points to a standard concentration–response curve [*Y* = 100/(1 + (X/EC50)^ – Hill)]. To assess the efficacy of the compound, the current amplitudes obtained at the maximal concentration of the compound were normalized to the maximal K_Ca_2.x/K_Ca_3.1 current in response to 10 μM Ca^2+^. Concentration–response curves were acquired from multiple patches for each data set. Each curve was fitted individually, which yielded the EC_50_ value for that curve. EC_50_ values are shown as mean ± SD obtained from multiple patches, and the number of patches is indicated by *n*.

The Student’s *t*-test was used for data comparison if there were only two groups. One-way ANOVA and Tukey’s post hoc tests were used for data comparison of three or more groups. Post hoc tests were carried out only if *F* was significant and there was no variance in homogeneity.

## Supporting Information Available

Electrophysiological recordings, cilia length measurements, intracellular Ca^2+^ signaling data, and amino acid sequence alignments (PDF)

## Abbreviations

K_Ca_2.1 channels: small-conductance Ca^2+^-activated potassium subtype 1 channels
K_Ca_2.2 channels: small-conductance Ca^2+^-activated potassium subtype 2 channels
K_Ca_2.3 channels: small-conductance Ca^2+^-activated potassium subtype 3 channels
K_Ca_3.1 channels: intermediate-conductance Ca^2+^-activated potassium channels
Ca_v_: voltage-gated Ca^2+^ channels
ER: endoplasmic reticulum
TRP: transient receptor potential
NO: nitric oxide
PKD2: polycystic kidney disease
ZLS: Zimmermann-Laband syndrome
