# Supplementary material for: Subtype-Selective Positive Modulation of KCa2.3 Channels Increases Cilia Length
Source: ACS Chem Biol. 2022 Aug 10;17(8):2344–54. doi: 10.1021/acschembio.2c00469 (PMC9396613; doi:10.1021/acschembio.2c00469)
Supplement: Supplementary file 1 — cb2c00469_si_001.pdf [file cb2c00469_si_001.pdf]

Supporting Information

Subtype-selective positive modulation of $K_{Ca}2.3$ channels increases cilia length

Young-Woo Nam [†,#], Rajasekharreddy Pala [†,#], Naglaa Salem El-Sayed [†], Denisse Larin-Henriquez [†], Farideh Amirrad [†], Grace Yang [†], Mohammad Asikur Rahman [†], Razan Orfali [†], Myles Downey [†], Keykavous Parang [†], Surya M. Nauli [*,†], Miao Zhang [*,†]

[†]Department of Biomedical and Pharmaceutical Sciences, Chapman University School of Pharmacy, Irvine, California 92618, USA

CORRESPONDENCE

* Surya Nauli

Department of Biomedical and Pharmaceutical Sciences

Chapman University School of Pharmacy

9401 Jeronimo Road

Irvine, CA 92618, USA

Email: nauli@chapman.edu

Tel: +1-714-516-5485; Fax: +1-714-516-5481;

* Miao Zhang

Department of Biomedical and Pharmaceutical Sciences

Chapman University School of Pharmacy

9401 Jeronimo Road

Irvine, CA 92618, USA

Email: zhang@chapman.edu

Tel: +1-714-516-5478; Fax: +1-714-516-5481

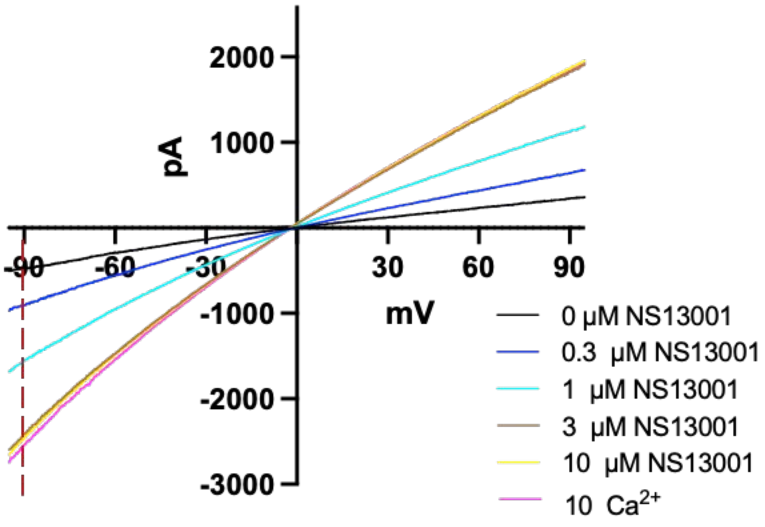

FIGURE S1. Representative human $K_{Ca}2.3$_WT channel currents in response to a positive modulator. The current amplitudes at -90 mV (indicated by a red dash line) in response to various concentrations of the compound were normalized to that obtained at a maximal concentration of the compound to construct the concentration-response curve.

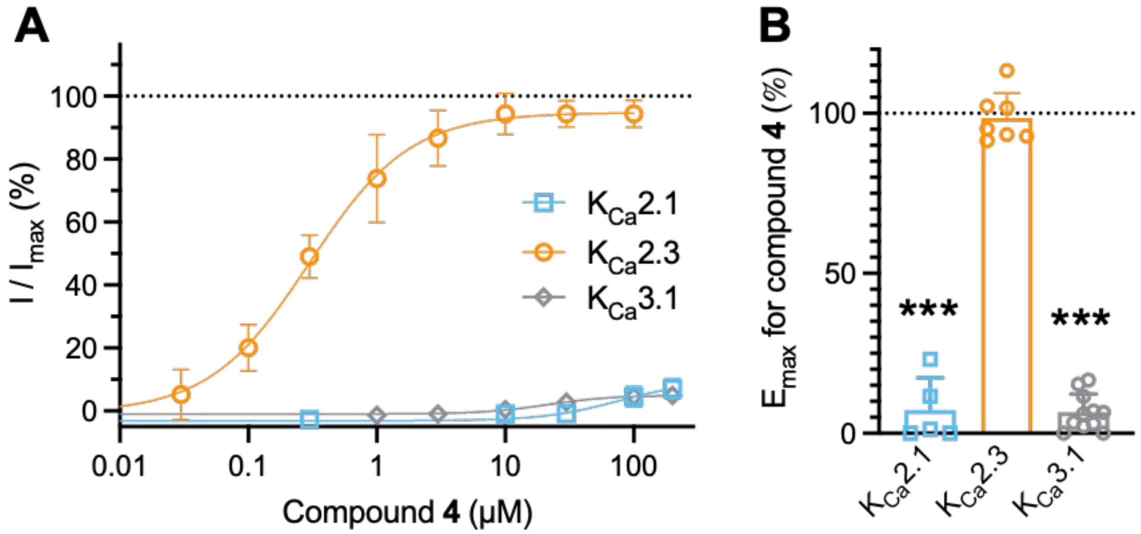

FIGURE S2. Lack of activity of compound 4 on human $K_{Ca}2.1$ and human $K_{Ca}3.1$ channel subtypes. (A) Responses to compound **4** of channel subtypes were normalized to the maximal currents induced by 10 µM Ca^{2+}. (B) E_{max} to compounds **4** of $K_{Ca}2.1$, $K_{Ca}2.3$ and $K_{Ca}3.1$ channels. The numbers of independent recordings are shown in parentheses for $K_{Ca}2.1$ (5), $K_{Ca}2.3$ (7) and $K_{Ca}3.1$ (11). Data are presented as mean ± SD. *** $P < 0.001$ compared with $K_{Ca}2.3$.

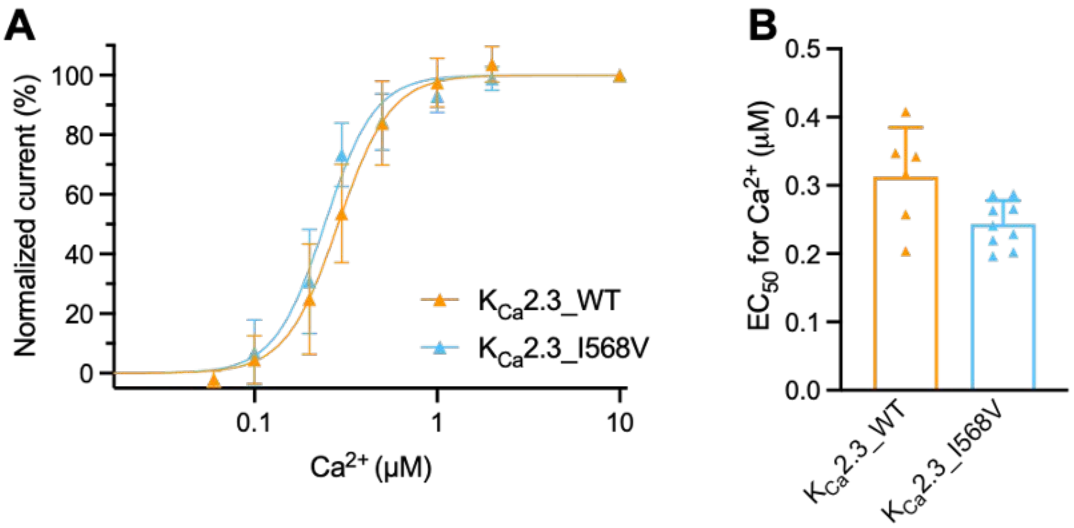

FIGURE S3. Mutations in the HA/HB helices of human K_{Ca} 2.3 channels did not change their apparent Ca^{2+} sensitivity. (A) Concentration-dependent activation by Ca^{2+} of the WT and mutant K_{Ca}2.3 channels. (B) EC_{50} values for activation by Ca^{2+} of the WT and mutant K_{Ca}2.3 channels. The numbers of independent recordings are shown in parentheses for K_{Ca}2.3_WT (6) and K_{Ca}2.3_I568V (9). Data are presented as mean ± SD.

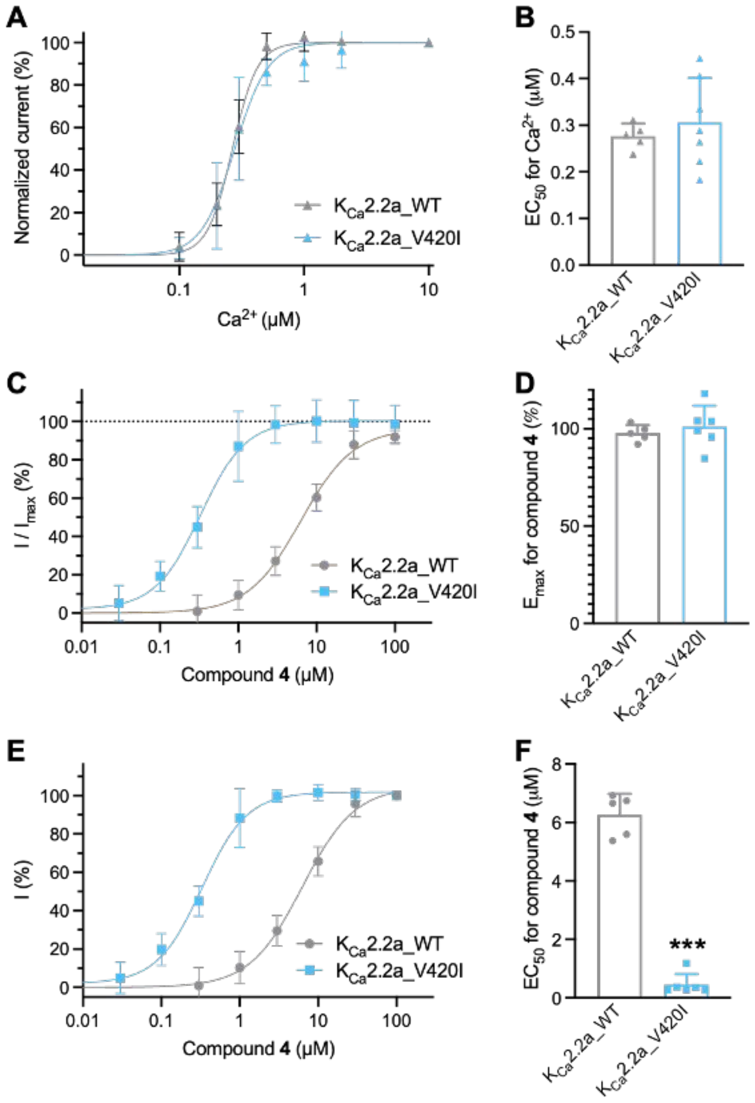

FIGURE S4. A mutation in the HA/HB helices of rat K_{Ca} 2.2a channels changed the sensitivity to compound 4. (A) Concentration-dependent activation by Ca^{2+} of the WT and mutant rat K_{Ca}2.2a channels. (B) EC_{50} values for activation by Ca^{2+} of the WT and mutant rat K_{Ca}2.2a channels. The numbers of independent recordings on Ca^{2+} are shown in parentheses for K_{Ca}2.2a_WT (5) and K_{Ca}2.2a_V420I (7). (C) Responses to compound **4** were normalized to the maximal currents induced by 10 μM Ca^{2+}. (D) E_{max} to compound **4** of the WT and mutant rat K_{Ca} 2.2a channels. (E) Potentiation by compound **4** of the WT and mutant rat K_{Ca}2.2a channels. (F) EC_{50} values for potentiation by compound **4**. *** $P < 0.001$ compared with K_{Ca}2.2a_WT. The numbers of independent recordings on compound **4** are shown in parentheses for K_{Ca}2.2a_WT (5) and K_{Ca}2.2a_V420I (6). Data are presented as mean ± SD.

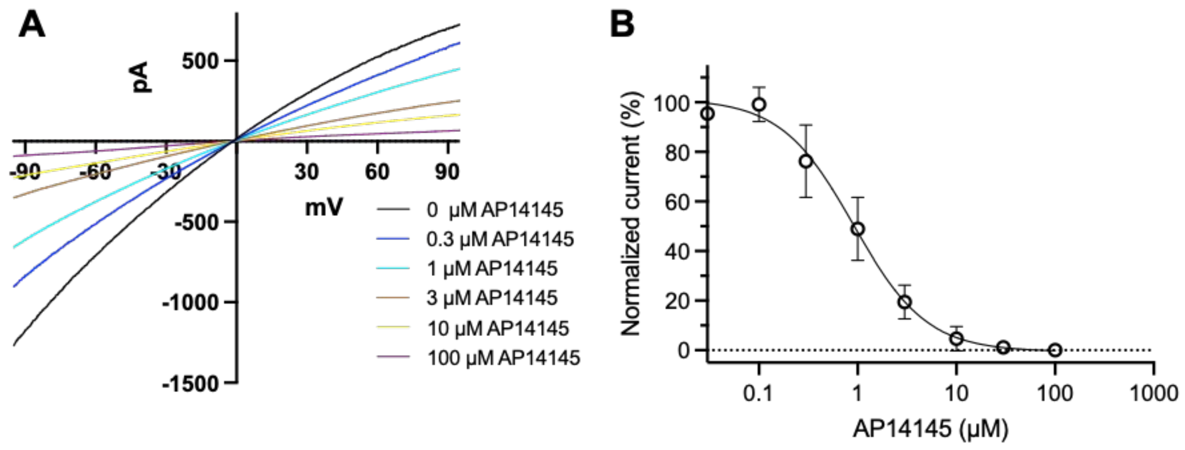

FIGURE S5. Inhibition of K_{Ca}2.3 channels by AP14145. (A) Representative K_{Ca}2.3_WT channel currents in response to AP14145. (B) Concentration-dependent inhibition of K_{Ca}2.3 current by AP14145. The number of independent recordings is 5. Data are presented as mean ± SD.

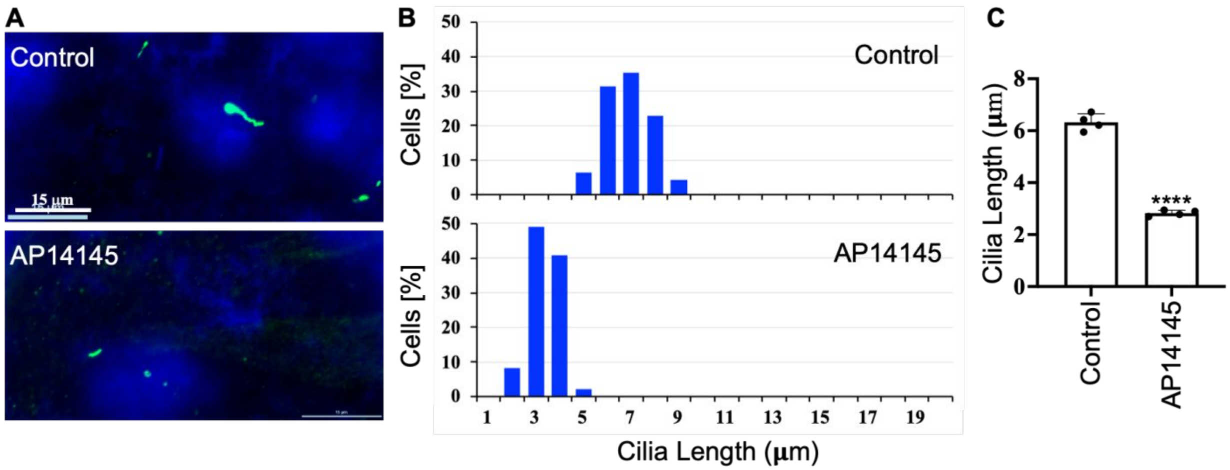

FIGURE S6. The effect of $K_{Ca}2.3$ channel inhibition by AP14145 on cilia length in ET cells. (A) Cells were stained with the antibody of a ciliary marker acetylated-α-tubulin (green) and a nuclear marker (DAPI; blue). (B) Cilia length was grouped in a discreet range and percent distribution was tabulated. (C) Cilia length is significantly shorter in cells treated with the negative modulator, AP14145 (20 µM). N = 50–70 for each slide preparation, and a total of 4 independent slides were used in each group. Data are presented as mean ± SD. **** $p < 0.0001$ compared to control.

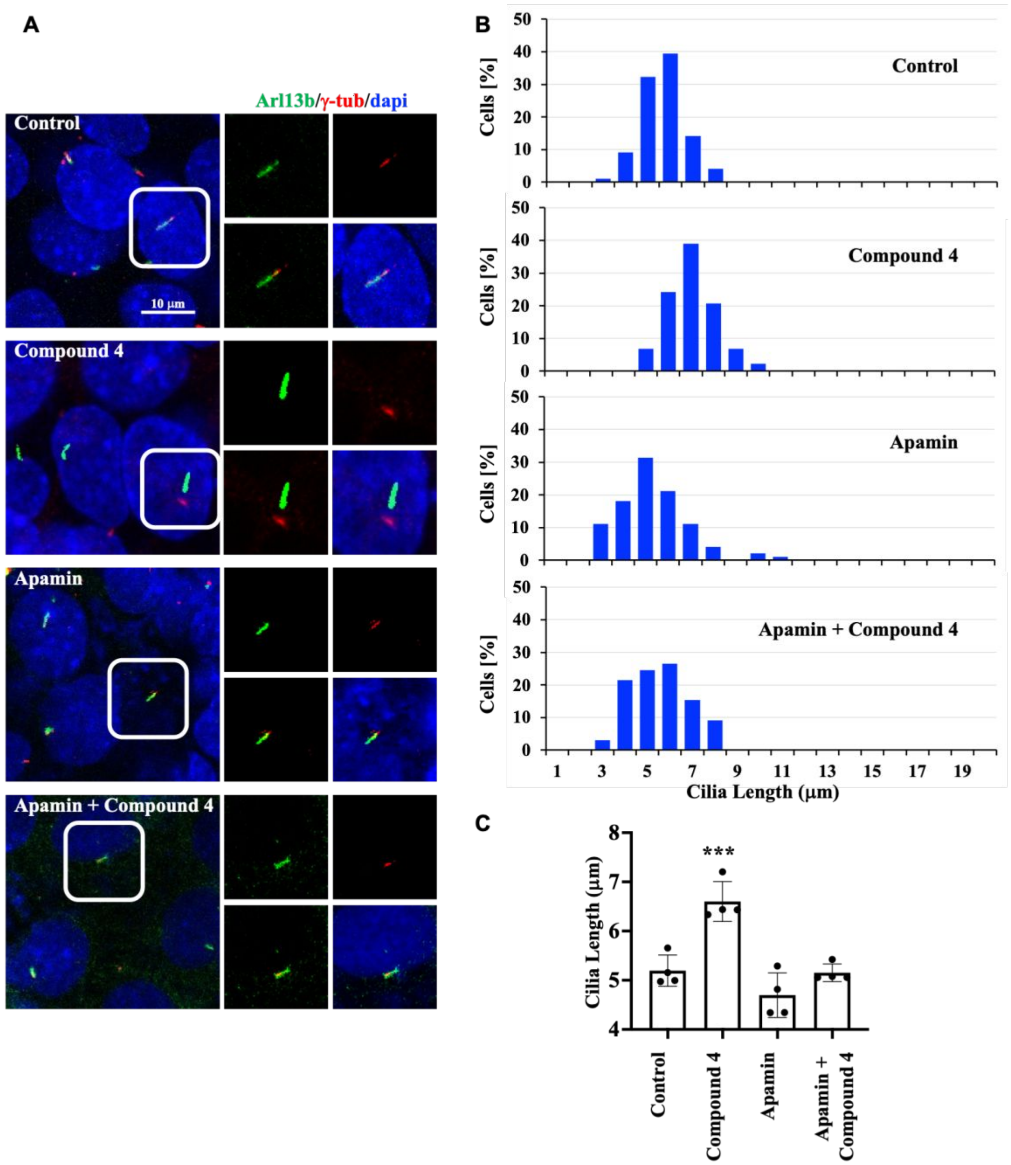

FIGURE S7. The effect of $K_{Ca}2.3$ channel activation by compound 4 on cilia length can be abolished by $K_{Ca}2.3$ channel blockade. (A) Cells were stained with the antibodies of ciliary marker Arl13b (green) and base of ciliary marker γ-tubulin (red), together with a nuclear marker (DAPI; blue). White boxes show larger image enrlargement to better visualize γ-tubulin. (B) Cilia length was grouped in a discreet range and percent distribution was tabulated. (C) Cilia length is significantly longer in cells treated with the compound **4** (20 µM). The elongation of cilia by compound **4** is abolished by the blockade of $K_{Ca}2.3$ channel with apamin. N = 50–70 for each slide preparation, and a total of 4 independent slides were used in each group. Data are presented as mean ± SD. *** $p < 0.001$ compared to control.

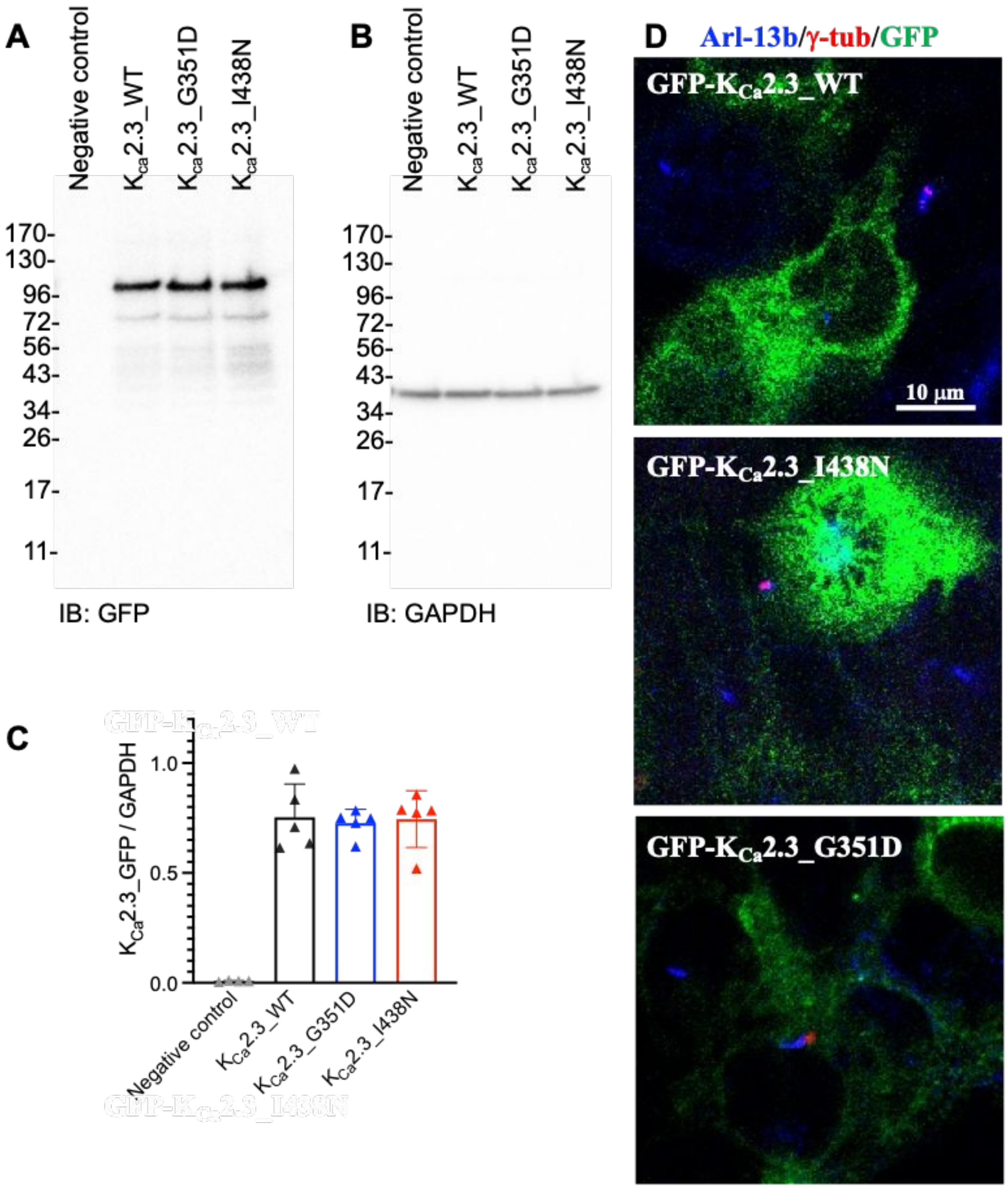

FIGURE S8. Comparable expression levels and localizations of mutant and WT K_{Ca}2.3 channels in ET cells. (A-C) Immunoblots of ET cells expressing GFP-tagged WT and mutant K_{Ca}2.3 channels, relative to a housekeeping protein GAPDH. (D) Cells were stained with the antibodies of ciliary marker Arl13b (blue) and base of ciliary marker γ-tubulin (red), together with GFP-tagged K_{Ca}2.3 channels (green). The number of independent experiments is 5.

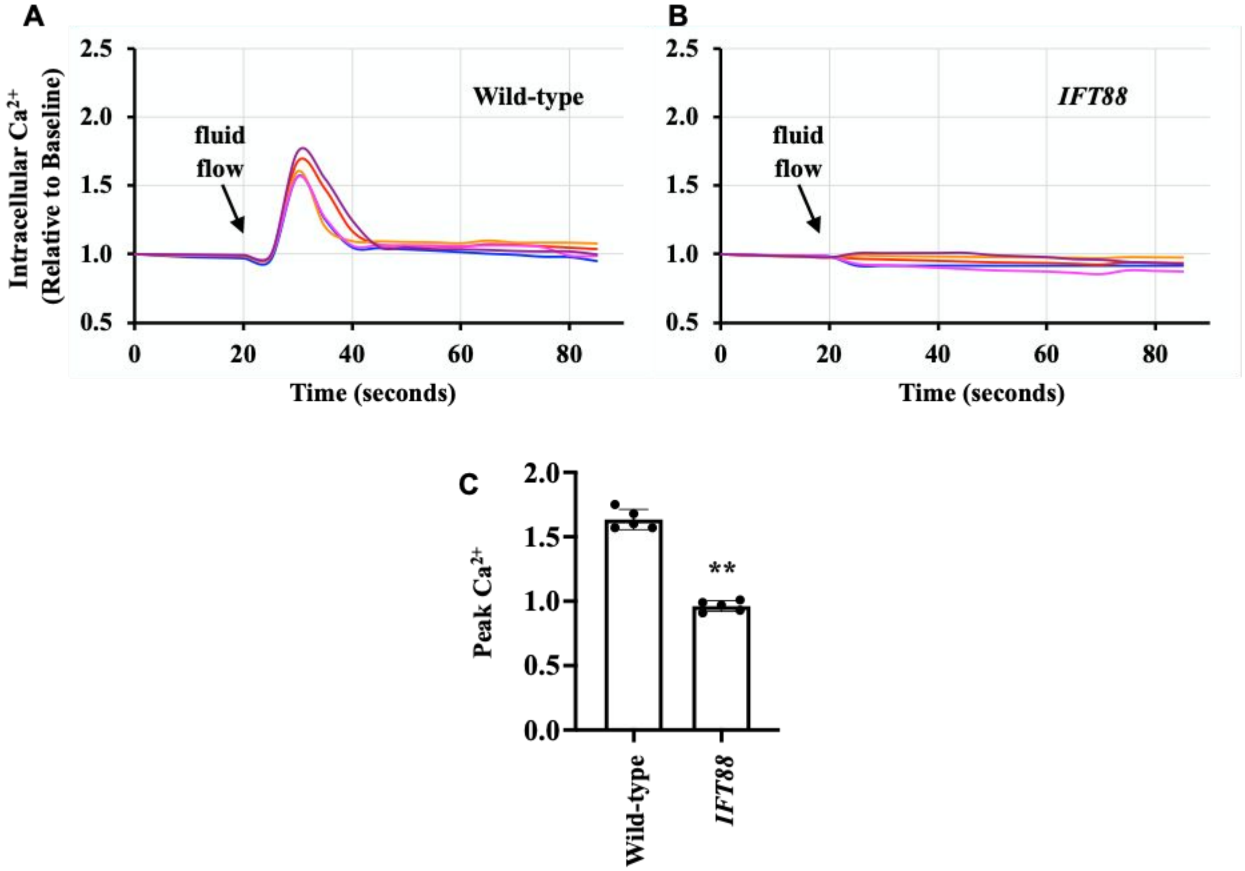

FIGURE S9. Lack of flow-induced cytosolic Ca^{2+} signaling in IFT88 knockout (KO) endothelial cells. Fluorescence Ca^{2+} measurements of (A) WT and (B) IFT88 KO endothelial cells. (C) Lack of Ca^{2+} transient in IFT88 KO compared with WT endothelial cells. The numbers of independent measurements are shown in parentheses for WT (5) and IFT88 KO (5). Data are presented as mean ± SD. ** $p < 0.01$ compared with control.

```
Human MSSCRYNGGVMRPLSNLSASRRNLHEMDSEAQPLQPPASVGGGG-GASSPSAAAAAAAAV 59
  Rat MSSCRYNGGVMRPLSNLSSSRRNLHEMDSEAQPLQPPASVVGGGGGASSPSAA----AAA 56
      ******************:********************* *** ********    **.

Human SSSAPEIVVSKPEHNNSNNLALYGTGGGGSTGGGG----GGGGSGHGSSSGTKSSKKKNQ 115
  Rat SSSAPEIVVSKPEHNNSNNLALYGTGGGGSTGGGGGGGGGGGGSGHGSSSGTKSSKKKNQ 116
      ***********************************    *********************

Human NIGYKLGHRRALFEKRKRLSDYALIFGMFGIVVMVIETELSWGAYDKASLYSLALKCLIS 175
  Rat NIGYKLGHRRALFEKRKRLSDYALIFGMFGIVVMVIETELSWGAYDKASLYSLALKCLIS 176
      ************************************************************

Human LSTIILLGLIIVYHAREIQLFMVDNGADDWRIAMTYERIFFICLEILVCAIHPIPGNYTF 235
  Rat LSTIILLGLIIVYHAREIQLFMVDNGADDWRIAMTYERIFFICLEILVCAIHPIPGNYTF 236
      ************************************************************

Human TWTARLAFSYAPSTTTADVDIILSIPMFLRLYLIARVMLLHSKLFTDASSRSIGALNKIN 295
  Rat TWTARLAFSYAPSTTTADVDIILSIPMFLRLYLIARVMLLHSKLFTDASSRSIGALNKIN 296
      ************************************************************

Human FNTRFVMKTLMTICPGTVLLVFSISLWIIAAWTVRACERYHDQQDVTSNFLGAMWLISIT 355
  Rat FNTRFVMKTLMTICPGTVLLVFSISLWIIAAWTVRACERYHDQQDVTSNFLGAMWLISIT 356
      ************************************************************

Human FLSIGYGDMVPNTYCGKGVCLLTGIMGAGCTALVVAVVARKLELTKAEKHVHNFMMDTQL 415
  Rat FLSIGYGDMVPNTYCGKGVCLLTGIMGAGCTALVVAVVARKLELTKAEKHVHNFMMDTQL 416
      ************************************************************

Human TKRVKNAAANVLRETWLIYKNTKLVKKIDHAKVRKHQRKFLQAIHQLRSVKMEQRKLNDQ 475
  Rat TKRVKNAAANVLRETWLIYKNTKLVKKIDHAKVRKHQRKFLQAIHQLRSVKMEQRKLNDQ 476
      ************************************************************

Human ANTLVDLAKTQNIMYDMISDLNERSEDFEKRIVTLETKLETLIGSIHALPGLISQTIRQQ 535
  Rat ANTLVDLAKTQNIMYDMISDLNERSEDFEKRIVTLETKLETLIGSIHALPGLISQTIRQQ 536
      ************************************************************

Human QRDFIEAQMESYDKHVTYNAERSRSSSRRRRSSSTAPPTSSESS 579
  Rat QRDFIETQMENYDKHVTYNAERSRSSSRRRRSSSTAPPTSSESS 580
      ******:***.*********************************
```

FIGURE S10. Amino acid sequence alignment of human and rat K_{Ca}2.2a channels. At the HA/HB helices (highlighted in green) where CyPPA interacts with, the similarity is 100%.
